# Experiences of internship nursing students in confronting ethical issues: a qualitative study

**DOI:** 10.1186/s12910-025-01254-w

**Published:** 2025-07-11

**Authors:** Tahereh Heidari, Leila Jouybari, Fariba Borhani, Zahra Sabzi

**Affiliations:** 1 Faculty of Nursing and Midwifery, Golestan University of Medical Sciences, Gorgan, Iran; 2 Medical Ethics and Law Research Center, Shahid Beheshti University of Medical Sciences, Tehran, Iran; 3 Nursing Research Center, Golestan University of Medical Sciences, Gorgan, Iran

**Keywords:** Internship, Nursing, Student, Ethics, Decision-making, Qualitative study

## Abstract

**Background:**

Advances in science and technology have turned the modern healthcare system into a challenging environment with many ethical dilemmas. Internship nursing students experience many ethical issues in the clinical environment. The present study explored the experiences of Iranian internship nursing students in confronting ethical issues.

**Methods:**

The present study is a qualitative inquiry with a conventional content analysis approach. A total of 15 internship nursing students (six females and nine males, semesters 7 and 8) were selected via purposive sampling with maximum variation from the nursing and midwifery faculty at Golestan University of Medical Sciences, northern Iran. Data were collected using semi-structured interviews over eight months. Graneheim and Lundman’s qualitative conventional content analysis approach was used for data analysis. MAXQDA version 2022 was utilized for data management. The trustworthiness of the data was ensured by four criteria given by Lincoln and Guba.

**Results:**

Qualitative data analysis led to 792 initial codes, which were reduced to 262 after removing duplicates. Subsequent analysis resulted in 7 sub-categories and 3 main categories, including “Insufficient Ethical Preparation,” “Inadequate Ethical Reasoning Skills,” and “An Ineffective Ethical Atmosphere in the Clinical Environment.” Ultimately, the main theme of “Limited Ethical Maturity of Students in Ethical Issues” was extracted.

**Conclusion:**

Internship nursing students enter the educational environment with inadequate preparation and limited ethical maturity, which often leads to passive behavior when faced with ethical issues. Considering the non-educational clinical environment and the identified issues in the academic structure, reviewing the curriculum of professional ethics in nursing in Iran seems necessary.

**Supplementary Information:**

The online version contains supplementary material available at 10.1186/s12910-025-01254-w.

## Background

Caring is a fundamental concept in the art of the nursing profession, and the ethical dimension of this art is regarded as an integral part of nursing practice [1, 2]. The ethical dimension of nursing practice emphasizes patient-centered care as reflected in the codes of ethics. Nurses must adhere to ethical principles such as respect for autonomy, justice, non-maleficence, beneficence, health maximization, efficiency, and proportionality [3, 4]. Along with the advancement of science and technology, issues such as limited resources, cultural and religious diversity among clients, an aging population, and shifting public expectations have turned the modern healthcare system into a complex environment fraught with ethical challenges that necessitate the development of sound ethical judgment and skills in health professionals [5, 6].

As the future workforce in the clinical environment, nursing students encounter challenging ethical issues that require ethical competency to identify and manage them to provide comprehensive care based on ethical principles [6, 7]. Ethical issues are situations in which profound ethical questions about “rightness” or “wrongness,” and what is good or bad, arise. Individuals must use this information as the basis for ethical decision-making and providing effective patient care [8]. The education and management of ethical issues are just as essential as acquiring knowledge and practical skills in nursing. Sometimes, attention to ethical principles takes precedence over technical aspects [4, 9]. The weakness of nursing students in understanding ethical principles and making appropriate ethical decisions may lead to negative consequences. These include unprofessional behaviors, improper therapeutic communication with patients, moral distress, and a disrupted professional identity. Ultimately, this can result in poor-quality nursing care [10, 11].

Previous studies have confirmed that nursing students and novice nurses lack the necessary knowledge and awareness to apply ethical practices in their skills effectively [12–14]. Due to limited awareness of key ethical documents such as the Declaration of Helsinki, many physicians and nurses often rely on lectures, textbooks, and academic journals for ethical guidance [15, 16]. Based on a study in Iran, 97% of nursing students encountered at least one ethical challenge, and 48% declared that their ethical challenges had not been resolved [17]. Prior studies have revealed that nursing students experienced ethical dilemmas in clinical settings, such as unprofessional communication with patients by healthcare staff, disregard for patient confidentiality, neglect of patient privacy, discrimination against patients based on socioeconomic status, disclosure of truth to terminally ill patients, euthanasia, and patient resuscitation [6, 18, 19]. Nurses also have expressed concerns about nursing students’ inability to make prompt and rational decisions while facing ethical dilemmas, attributing this to a lack of essential nursing skills such as moral awareness and sound judgment [20, 21].

Nursing students in Iran complete 130 theoretical and practical course units during their 4-year bachelor’s degree program. They enter the internship course in the last year of education (semesters 7 and 8). In the internship course, students should pass 19 credits in all clinical departments without the direct supervision of clinical educators and under the supervision of clinical nurses and head nurses. They are obligated to complete different work shifts similar to other nursing personnel. They pass 1.5 credits of Professional Ethics in Nursing (in semester 3) and a four-hour workshop (in semester 5) before the internship [22]. They lack a dedicated course on professional ethics in the clinical setting. Still, they may encounter ethical issues to a limited extent in their other courses or clinical settings, and the professors clarify those ethical issues to them. So, internship students continuously experience many ethical problems, and their ethical behavior can facilitate complex clinical judgments; however, despite the importance of this issue, the content of ethics education in nursing degrees in Iran is inconsistent, highly variable, and lacks a basic structure.

The lack of a practical and efficient approach to nursing ethics education confuses nursing students and prevents them from acquiring knowledge and skills related to ethics [23]. Based on the prior studies, conducting a more in-depth investigation into the experiences of Iranian nursing students to address ethical issues within the clinical setting appears imperative. Compiling appropriate course content for ethics education requires understanding the possible gap between clinical practice and academic concepts.

To gain a comprehensive understanding of internship nursing students’ actual experiences in addressing ethical issues, employing a qualitative approach is advantageous. Qualitative research is an appropriate method for understanding and discovering the perceptions and experiences of participants without interfering with the researcher’s assumptions about the study process [24, 25]. Based on the constructive or naturalistic paradigm, reality is not a unique and fixed issue and is created by the mind depending on the context [26, 27]. Therefore, we chose to use a naturalistic framework due to the inherently subjective, complex, and multidimensional nature of the experiences of nursing students during their internships when addressing ethical issues. Regarding the importance of ethics education in nursing and the exposure of internship students to ethical issues, the present study was conducted to explore the “experiences of Iranian internship nursing students in confronting ethical issues.

## Methods

### Study design

This qualitative study employed the conventional content analysis approach as a systematic method to explore a phenomenon in-depth and to investigate people’s experiences on a specific issue [28]. The qualitative content analysis approach was used [29] to explain the experiences of internship nursing students addressing ethical issues.

### Data collection

Data were collected through in-depth and face-to-face semi-structured interviews from September 2023 to April 2024. We have not published the interview guide before this project. We recruited participants via purposive sampling to ensure maximum diversity, including variations in gender, academic semester, internship experience across different wards, and native or non-native status (Is the student a Golestan province resident or not? ). Purposive sampling provided comprehensive and in-depth information. The inclusion criteria consisted of willingness to participate in the study and internship nursing students in their 7th or 8th semester who can articulate their experiences in patient care. Participants were not paid for the interviews and participated in the study voluntarily.

The interviews were conducted after scheduling with the participants regarding the time and place. The first author, who trained in qualitative studies, conducted the interviews in a suitable and quiet environment at one of the educational hospitals or the Faculty of Nursing and Midwifery. First, a pilot interview was conducted to adjust the interview questions. Persian was used to conduct the interviews, and participants also used this language to answer the questions.

The first participant was recommended by a university faculty member who worked in a clinical setting. This faculty member supervised nursing students during their internships in various departments, maintaining close interactions with students, head nurses, and hospital administrators. This participant was selected because of her honesty in reporting errors and ethical issues in the clinical setting, as she would promptly and honestly report ethical issues and problems to the clinical faculty member. Additionally, the nursing staff and head nurses underscored the ethical practice of this internship student. This selection was likely the best choice to achieve the study’s objectives in the primary step. After scheduling and explaining the study’s objectives, the interview was conducted with the participant in a room at the Faculty of Nursing and Midwifery.

Each interview lasted between 45 and 60 minutes, depending on the participant’s cooperation. During all the interviews, the participants’ privacy was guaranteed, and each interview was conducted individually. The interview started with a general question: Can you describe one of your work shifts in the clinical environment (internship) for me? For example, how do you take care of the patient from the moment you enter the ward? During your internship in various hospital wards, have you encountered any issues that you found to be of ethical significance? Please describe your experience. Have you encountered situations where you had to choose between several options? such as whether to be honest with a patient or not. What would you do if you encountered it? Please share your experience with me.” Based on the participants’ responses, the interview proceeded with probing questions such as, “Can you explain further and provide an example?” to clarify their answers when necessary.

We interviewed 15 participants and conducted 20 interviews. Five additional interviews were conducted with Participants 2, 3, 5, 7, and 15 to clarify unclear points and details in their initial interviews. The first author, a PhD student and a clinical instructor with extensive clinical experience, was trained in qualitative studies and conducted the interviews. Data collection continued until data saturation. Saturation indicates that no new codes can be identified, and the collected data comprehensively reflect the phenomenon under investigation [30]. The present study reached saturation after the 13th interview, and two additional interviews were conducted to ensure data saturation. Upon reviewing all potential codes and categories, no new codes or categories were added to the existing ones. Consequently, the data collected can be deemed comprehensive and representative of the phenomenon under investigation. The first author recorded the participants’ interviews using a Samsung J5 mobile phone, transcribed with Microsoft Office Word software, and saved in an encrypted file on her computer.

### Data analysis

Data analysis was done using the conventional content analysis approach of Granheim and Lundman (2004) [29]. The data analysis process was initiated by the first author, a PhD student and the corresponding author, who holds a PhD in Nursing and is an Associate Professor at the School of Nursing of Golestan University. Subsequently, they consulted with advisors who also hold PhDs in Nursing and are professors at nursing schools at Golestan University of Medical Sciences and Shahid Beheshti University of Medical Sciences, to reach a consensus on the codes, subcategories, main categories, and final theme.

The analysis process included several steps, starting with the first author reading each transcribed interview multiple times to immerse in the data and understand each participant’s story comprehensively. In the next step, meaning units such as words, sentences, or paragraphs pertaining to the study goal were identified. These meaning units were then condensed to shorten the text while maintaining the main content. A code was assigned to each condensed meaning unit. In this study, a maximum of initial codes emerged (792 initial codes), which were reduced to 262 after removing duplicates. The codes were compared based on their differences and similarities, and grouped into the initial 7 sub-categories. By continuous comparison, similar sub-categories were combined, resulting in the formation of three main categories. Then, the main categories were combined to reveal the main theme and the interviews’ hidden content. Ultimately, the theme of “Limited Ethical Maturity of Students in Ethical Issues” emerged.

MAXQDA 2020 software was used to manage and organize data. Data analysis was conducted concurrently with data collection. After each interview, the recordings were transcribed verbatim and input into MAXQDA software. Using this software, initial codes were generated and exported. After each interview, the newly extracted codes were compared with those from previous interviews, and duplicate codes were eliminated. The initial codes were manually analyzed to identify sub-categories, main categories, and final themes.

### Rigor and trustworthiness

The trustworthiness of the data was ensured by applying four criteria given by Lincoln and Guba (2007), including credibility, dependability, confirmability, and transferability of data [31].

Before beginning the study, a pilot interview was conducted to gain an initial understanding of the subject. To establish credibility, the researcher engaged in prolonged immersion in the data, dedicating sufficient time to data collection and analysis, and continuously comparing data sets. To ensure the dependability of the extracted codes, they were shared and reviewed by participants, then adjusted based on their feedback. To achieve the confirmability, peer checks and audit trails were employed. The extracted codes and categories were peer-reviewed by experts, and the findings were revised according to their suggestions. To meet the criterion of transferability, participants were selected with a wide range of diversity. The research process and findings were carefully described and detailed to address this concern, facilitating the transfer and replication of findings in similar studies. In summary, these measures were implemented to ensure the rigor and trustworthiness of the data.

## Results

### Characteristics of participants

The participants included 15 undergraduate nursing students undergoing internship courses (semesters 7 and 8) at the educational hospitals of Golestan University of Medical Sciences, in the north of Iran. Of the participants, 60% were male, with a mean age of 23.73 years. The average duration of the interview with the participants was 56.33 min. The demographic characteristics of the participants are presented in Table 1.

**Table 1:** Demographic characteristics of the participants

characteristic		*N* (%)
**Sex**	Male	9 (60)
	Female	6 (40)
**Semester**	Semester 7	5(33.3)
	Semester 8	10 (66.7)
**Residence status**	Native	7(46.67)
	Non-native	8 (55.33)

### Qualitative findings

A central theme and three main categories were identified through data analysis. The central theme is the “Limited Ethical Maturity of Students in Ethical Issues,” representing the fundamental nature of other categories. The final theme encompasses three main categories: “Insufficient Ethical Preparation,” “Inadequate Ethical Reasoning Skills,” and “An Ineffective Ethical Atmosphere in the Clinical Environment.” The central theme, main categories, and corresponding sub-categories are presented in Table 2.

**Table 2:** The theme, main categories, and sub-categories of internship nursing students’ experiences in facing ethical issues

Theme	Main Category	Sub-Category
**Limited Ethical Maturity of Students in Ethical Issues**	**Insufficient Ethical Preparation**	Ineffectiveness of Ethics Courses in Addressing Ethical Challenges in the Clinical Setting
		Feeling Incapable of Handling Ethical Issues Due to Student Status
	**Inadequate Ethical Reasoning Skills**	Limited Identification of Instances of Deviation from Ethical Standards
		Student Reflection in Challenging Situations
		The Passive Behavior in Ethical Issues
	**An Ineffective Ethical Atmosphere in the Clinical Environment**	Inefficient Professional Communication of Nurses with Other People
		Non-Educational Environment in Terms of Professional Ethics

We included direct quotes from internship nursing students to further elaborate on these categories.

### Insufficient ethical Preparation

Due to their similarity and compatibility, two sub-categories: “Ineffectiveness of Ethics Courses in Addressing Ethical Issues in the Clinical Setting” and “Feeling Incapable of Handling Ethical Issues Due to Student Status” were grouped under the higher-level abstract “Insufficient Ethical Preparation”.

#### Ineffectiveness of ethics courses in addressing ethical issues in the clinical setting

Many participants highlighted the inapplicability of the nursing professional ethics course in the undergraduate curriculum as a significant issue. They mentioned issues such as teaching theory-based ethical topics in nursing professional ethics courses and the inability of some professors to sensitize students to ethical principles. One of the participants stated:

“The professor who taught us had a monotonous voice, his class was boring, or another professor who only read articles and mentioned things about euthanasia… Some professors have good knowledge in the field of ethics, but they don’t express it well“(P: 15, Male, Semester 8).

Another student pointed out the lack of summarization of the information and scenarios presented in the nursing professional ethics class.

“In the nursing ethics course unit, the professors would present a series of scenarios, and we would discuss them. Well, you know, everyone had their own opinion, but in the end, there was no conclusion to that topic, and before it was concluded, the class would end. In the next session, when the professor arrived, he did not discuss the previous topic anymore and began a new subject” (P:3, Female, Semester 8).

Another participant also stated:

“They taught by PowerPoints and would present a series of scenarios. For example, they discussed topics that are not legal in our country, such as euthanasia. They would present scenarios such as: if a patient requests not to receive CPR, will you accept it? Then they would ask for our opinions instead of giving a definitive answer themselves” (P: 4, Female, Semester 8).

Some participants pointed out that the nursing professional ethics course was not very important to them. One of the participants expressed:

“Well, I didn’t pay much attention to the ethics course because I didn’t think it was crucial… we also knew that those four ethical principles are important in Objective Structured Clinical Examination (OSCE), so we focused on them” (P: 7, Male, Semester 7).

The participants also mentioned the inability of some clinical educators to manage ethical challenges in the clinic. One participant stated the following:

“I witnessed a mistake made by a nurse in the ward, but I didn’t tell the head nurse, because we don’t have that much acceptance in front of them, and they don’t pay much attention to the student’s statements. I reported to my clinical educator, but he just said it was a mistake. However, I didn’t see him give a reminder to the head nurse or the nurse (P: 12, Male, Semester 7).

#### Feeling incapable of handling ethical issues due to student status

Many participants noted that the healthcare team in the clinical environment often overlooks their opinions. Additionally, students feel they lack the authority to express their views and perform independent procedures in this setting. It seems that students often lack the necessary self-confidence and moral courage to express their opinions and report errors during their internship period. They also stated they have no responsibility in the patient care process and that the nurse in charge should be accountable.

One participant expressed:

“The fact that I have no responsibility here in the hospital as an intern is completely normal. I am an internship student, and even the responsibility of the patient given to me is with the nurse in charge” (P2: Female, Semester 8).

The participants also mentioned concerns about the healthcare team’s disregard for their opinions and the limited authority they felt within the clinical environment, which contributed to their hesitation in reporting ethical challenges they observed in the clinical setting. One participant said as follows:

“We often have limited acceptance of them. For instance, I once informed the nurse in charge that a patient was in pain, but she ignored my repeated warnings (I reported the patient’s pain three times). I told her, “You need to prioritize now. You need to manage the patient’s pain now.” She became angry and reported me to the head nurse and supervisor, resulting in two additional shifts as a penalty for me. As a student, can I administer analgesics to the patient myself? " (P: 14, Male, Semester 8).

Another participant said:

“One experience that has stuck with me involved a patient who had been diagnosed with cancer. The nursing staff instructed us: “Do not disclose any diagnostic information to the patient.” One day, when I entered the patient’s room to administer his medications, he asked, “What did the doctor say about my condition?” In that moment, I felt confused and didn’t know how to respond. I understand that the patient has the right to know about their diagnosis, but I didn’t know how to tell him about his diagnosis. I told him, “Ask your doctor,” and quickly left the room (P: 14, Male, Semester 8).

Another participant mentioned the differences between our culture and Western culture in some issues:

“We had a patient with gastric cancer who was unaware of his diagnosis. A consultation was requested for him, although I can’t recall what consultation. When the consulting doctor arrived, he asked the patient, “Do you know you have cancer?” and the patient realized it. Later, when I went to administer the prescribed medication, the patient angrily declared, “I’m leaving. I won’t allow any further treatment. Even if my condition worsens and I’m dying, refuse any intervention for me.” I reported this to the nurse in charge, who advised me to wait and try again. Upon returning after half an hour, the patient still adamantly refused, stating, “I made it clear; I don’t want any treatments.” Feeling conflicted, I remembered our professors emphasizing respecting patients’ autonomy. They also mentioned that our culture differs from Western cultures on these issues. It was even discussed in class what to do if a patient didn’t resuscitate, but no definitive answer was provided. In that moment, I truly didn’t know what to do. What should I say to the patient?” (P: 10, Male, Semester 8).

### Inadequate ethical reasoning skills

The sub-categories of “Limited Identification of Instances of Deviation from Ethical Standards,” “Student Reflection in Challenging Situations,” and **“**The Passive Behavior in Ethical Issues” were grouped under the higher-level abstract entitled “Inadequate Ethical Reasoning Skills.”

#### Limited identification of instances of deviation from ethical standards

Internship students talked about their experiences with nurses who violated ethical principles in patient care, particularly regarding respect for patients’ lives and dignity. One participant mentioned:

“We had a patient with liver cancer that had severe metastases in the end stage. When the patient had a cardiac arrest, the CPR code was announced, just a nominal mode of giving the resuscitation code, and everyone entered the room. Then I was also in the room, and the nurses and other members of the CPR team did not resuscitate the patient. I felt bothered that the nurse was not taking any action, and it was frustrating to hear the healthcare team telling jokes and laughing nearby. Isn’t it true that the last sense to lose is hearing? This means the patient still heard what they were telling. They did not even show respect to the patient during CPR” (P: 11, Male, Semaeter7).

Some of the participants’ perceived ethical conflicts were also related to nurse-patient relationships.

“We had a patient, a young girl who engaged in high-risk sexual behaviors… Then, during the handover, the nurses were saying that it’s unclear what she does, it’s unclear how many partners she’s had, and her mother is even worse!!! The patient was also near the nursing station and overheard. She was somehow depressed. “They didn’t care about confidentiality at all” (P: 3, Female, Semester 8).

Another participant mentioned the disclosure of patient information:

“Once I went to the college cafeteria for lunch, I saw some of my classmates talking about an old man in the internship department where I attend. They were talking in detail… the old man, whose bed was by the window, was speaking very irrelevantly. His words were funny and… I was surprised and realized who they were talking about… You see, the students thought confidentiality was limited to not revealing the patient’s name” (P: 10, Male, Semester 8).

Some participants pointed to the observation of non-compliance with standards related to the clinical procedures performed by nurses. A participant said:

“One patient with a hiatal hernia required a change of dressing over the surgical site. His dressing was such that sterile gas had to go into that cavity, and now it had to be very sterile. The nurse initiated the dressing change without wearing sterile gloves. Now he would leave the tweezers outside the dressing pack and continue dressing with the same non-sterilized tweezers. The head nurse observed the procedure from within the nursing station, and I accompanied the nurse during the dressing change. The nurse asserted that sterile gloves were not required for this procedure, despite the need to maintain sterility. I also had to wear latex gloves” (P: 1, Female, Semester 8).

#### Student reflection in challenging situations

Participants stated that when faced ethical challenges or when they observe nurses’ lack of attention to ethical principles or their mistakes, they analyze the situation and are cautious in reporting errors. One participant said:

“If I notice a nursing error, I will first consider the nurse’s work experience. If the nurse has extensive experience, I will seek their assistance, but if the nurse is a novice or has limited experience, I will address the mistake directly with them. Additionally, if I observe a potential error made by a nurse, I might ask another nurse, for example, “Do you think that action was appropriate?” That has a question form and is indirect” (P: 2, Female, Semester 8).

Another participant said that if he makes a mistake, reporting it depends on the reaction of the nurse:

“If I make a mistake, the report depends on the reaction of the nurse in charge. For example, in a clinical ward, I administered cefazolin to the wrong patient, but knowing the nurse would be angry, I did not inform her. However, if I am sure that the nurse will not react negatively and reporting the error will be beneficial, I will do so. In another ward, I prepared insulin to administer to the patient. I realized that I had drawn 40 units, which felt excessive. I felt in doubt and returned to the nurse. I requested him to recheck the patient’s medication card. Upon review, I discovered the correct dosage was 4 units and modified the error**”** (P: 15, Male, Semester 8).

Another student mentioned hesitating before taking action:

“The nurse instructed me to check a patient’s blood sugar, which was 250, and I think that was slightly higher. When the patient asked me, I hesitated and then reported it as 200 to avoid distressing him, based on prior experience in another department. As my primary duty is to benefit the patient and avoid causing harm, I chose not to reveal the exact BS to prevent stress. So, I provided a number close to the actual BS to ensure he would not resist necessary medication” (P: 11, Male, Semester 7).

#### The passive behavior in ethical issues

Many participants chose to remain passive when facing ethical dilemmas and simply acted as observers. A lot of them preferred to stay silent rather than become whistleblowers. One participant shared the following experience:

“When we are in a clinical department, we follow the routines established there. I have noticed practices that deviate from what our clinical educators and professors taught us during our training. I pointed out one of these discrepancies to a nurse, and she responded by saying that such practices are based on ‘book knowledge.’ She explained that with work experience, I would learn that some procedures are not always necessary. So, I have been working according to the routine of that department” (P: 13, Female, Semester 7).

Another participant pointed out the inequity in patient care in some clinical wards:

“I encountered injustice in some wards. In a ward, a patient acquainted with the head nurse received constant attention and special care, while another patient, an addict, often complained of pain and was mostly ignored by the nurses. I told the nurse that the patient was in pain and had a physician’s order for analgesics. She said, “Leave it out. Just inject him with sterile water.” I said again that the patient had an analgesic order, but she insisted, “I’m telling you to inject him with sterile water; he will calm down”. I did not argue further and administered her order” (P: 9, Female, Semester 8).

However, a limited number of participants also mentioned their assertive behavior when faced with ethical challenges or when nurses failed to meet care standards. They took appropriate action by relying on their knowledge. A participant said:

“Once a patient was in pain, I informed the nurse several times. Her response was dismissive, telling me to “let it go”. However, I noticed that the patient continued to complain of pain. Upon reviewing the patient’s medical records, I discovered that they were prescribed an Apotel ampoule for pain management as needed (PRN). With this information in mind, I administered the Apotel to the patient as per the physician’s instructions. I retrieved the Apotel ampoule from the patient’s medication box without informing the staff, as I was confident in the correctness of my actions” (P: 4, Female, Semester 8).

### An ineffective ethical atmosphere in the clinical environment

The sub-categories of “Inefficient Professional Communication of Nurses with other People” and “Non-Educational Environment in Terms of Professional Ethics” were grouped under the higher-level abstract main category titled “An Ineffective Ethical Atmosphere in the Clinical Environment” due to their similarity and compatibility.

#### Inefficient professional communication of nurses with other people

One of the challenges observed or experienced by internship students was the ineffective communication of nurses, which encompassed interactions between nurses and patients, nurses and caregivers, as well as nurses and internship students. The participants’ quotes regarding this issue are presented below.

“There was a patient’s companion who was a very respectable individual. He tried to ask each question only once and was very calm. At one point on the informed consent form that he was supposed to sign, he forgot to write his name. The nurse said loudly and disrespectfully, “Good thing I told you to write your name. Don’t you understand what language I’m speaking to you **in?** That poor man replied: “I’m sorry” (P: 3, Female, Semester 8).

Another participant said:

“We had a conscious patient in the ward, who was an addict. He was aggressive and cursing, but the head nurse of the ward should have been a bit more patient. When the patient cursed, she insulted him again…” (P: 4, Female, Semester 8).

The nurses did not establish respectful communication with the internship nursing students. Sometimes, this disrespectful behavior occurred in the presence of the patient. A participant said:

During a busy shift in the emergency department, multiple patients required an electrocardiogram (EKG), and I inadvertently placed the leads in the wrong position. Following this mistake, the ward nurse shouted, “You’re in your seventh semester and still don’t know how to place EKG leads?” I explained that I had been distracted by the high volume of activity. It was very disrespectful behavior, especially in front of the patient“(P: 13, Female, Semester 7).

#### Non-Educational environment in terms of professional ethics

Internship students expressed their experiences of inconsistency between the principles taught and the realities of nursing practice in the clinical setting. They pointed to issues such as the discrepancy between theory and clinical practice, the non-compliance of standards related to clinical skills by nurses, and assigning clinical procedures to internship students without the supervision of the responsible nurse. The quotes of some participants are presented as follows.

“ I also saw in some departments, for example in the surgery ward, the nurse noted in the nursing report: The patient was taught or the patient’s change position was done, but I witnessed that the nurse did not do such a thing from morning and only recorded it in the report” (P: 12, Male, Semester 7).

Another participant said in this regard:

“During a night shift, I observed that the nursing staff did not consider the timing and were administering two medications at different times simultaneously. I asked whether it was right to give medications at the same time. The nurse responded that our ward lacked sensitivity compared to specialized units like the CCU, and that the medications prescribed here were mainly antibiotics, analgesics, and routine treatments. Nonetheless, I was advised not to perform such actions in the presence of the head nurse and to refrain from informing the head nurse that this practice occurred during the night shift” (P: 5, Male, Semester 7).

Some participants also raised the limited opportunities for nursing students to practice or execute medical procedures during internship training as another challenge in the clinical environment. A participant said:

“He does procedures like NGT by himself, he doesn’t let us touch and try at all. He is afraid that something will happen. In addition, he doesn’t devote time to train us in such procedures” (P: 6, Male, Semester 8).

Some participants witnessed inappropriate safe discharge processes and insufficient attention from nursing staff regarding patient education. One of the participants mentioned:

“On the orthopedic ward, I didn’t see the nursing staff teach the patient at discharge, just handing a routine discharge sheet to the patient or caregiver, which is usually an illegible sheet. Just a few days ago, the nurse gave me the training sheet to teach him while discharging the patient. I said, “Tell me what you wrote. “At least I trained him. He told me to give that sheet to him; there’s no need to explain” (P: 2, Female, Semester 8).

## Discussion

The present qualitative study investigates the experiences of Iranian nursing internship students facing ethical issues in clinical settings. Our study results revealed that internship students have limited ethical maturity when faced with ethical issues, affecting their ability to manage ethical dilemmas. Ethical maturity is crucial for nurses to make independent and effective decisions in complex clinical situations. By relying on ethical principles, nurses can achieve optimal solutions. Nursing students, however, often lack the experience, talent, and courage to handle ethical dilemmas effectively, indicating a lower level of moral maturity compared to professional nurses [32, 33].

The theme of “limited ethical maturity” was abstracted from three main categories: “Insufficient Ethical Preparation,” “Inadequate Ethical Reasoning Skills,” and “Ineffective Ethical Atmosphere in the Clinical Environment.”

### Insufficient ethical Preparation

One feature of the main category, “Insufficient Ethical Preparation,” was the Ineffectiveness of Ethics Courses in Addressing Ethical Challenges in the Clinical Setting. Before entering this course, students are required to have a solid grasp of ethical issues and conflicts. This requires understanding the theoretical framework of nursing professional ethics principles, alongside receiving practical guidance from clinical instructors in real-life clinical settings. Students need to be sensitized to the ethical dimension of patient-centered care. To achieve this goal, professors and clinical instructors should strive to make the professional ethics unit engaging for students by presenting interesting and challenging scenarios that align with ethical principles. However, our study’s findings suggest that this objective was not met in professional ethics in nursing credit, primarily due to factors such as faculty teaching styles, clinical instructors’ inability to address ethical issues, and students’ low regard for the ethics module. The previous studies also emphasized the insufficient readiness and challenges of students to enter the clinical environment, and considered that the lack of necessary knowledge and skills before entering the clinical environment disrupts their learning process in the clinical setting, which is in line with this study [13, 34, 35].

Consistent with the results of our study, Borhani et al. (2012) revealed that ethics education in the Iranian nursing education system faces several challenges. These challenges include: a lack of coherent ethical content, reliance on traditional pedagogical approaches, a shortage of experienced and trained instructors in ethics education, inadequate contextualization of educational materials to students’ clinical realities, a focus on Western culture in ethics education, and students with weak interpersonal communication skills [36]. Ethics education should be considered as a valuable opportunity to learn how to regulate emotions and understand the intricacies of moral dilemmas, which notably impact students’ decision-making when confronted with ethical issues [7, 14, 37]. Therefore, it seems necessary for instructors to employ varied teaching strategies and appropriate course content to promote students’ ethical decision-making ability and moral courage in confronting ethical dilemmas.

Another feature related to the above main category was “Feeling Incapable of Handling Ethical Issues Due to Student Status.” The results of other investigations also emphasized the inferiority complex and the lack of authority of students in clinical environments [17, 34]. The diminished self-confidence experienced by internship nursing students within clinical environments is an influential factor leading to fear and anxiety, which affects their professional communication skills, resulting in low-quality nursing care [38, 39]. The lack of ethical courage among nursing interns hinders their professional identity development. By employing innovative pedagogical methods like simulation-based training, ethics educators will help cultivate ethical courage and enhance interns’ self-esteem before their internships.

### Inadequate ethical reasoning skills

One feature related to the main category of inadequate ethical reasoning skills was “Limited Identification of Instances of Deviation from Ethical Standards.” Our results showed that internship students can identify limited instances of deviation from ethical standards. These ethical issues were nurses’ weak attention to the patient’s confidentiality and privacy, neglect to obtain the principles of informed consent from the patient, the patient’s request not to resuscitate, the patient’s unawareness of his diagnosis due to the family’s request, and the family’s request upon not to resuscitate the patient with cancer which is consistent with the findings of previous studies [14, 40–42].

In this regard, it is worth noting that students often feel helpless when faced with a conflict between respecting the principle of patient autonomy and their personal and religious values. They may have difficulty making decisions in such situations, especially in Iranian culture, where issues such as assisting a patient in dying or practices like euthanasia are not widely accepted. Iranian people have religious attitudes and beliefs that significantly influence various aspects of their lives, including the healthcare system and the nursing profession. Showing respect for ethical values and valuing others are essential in Iranian culture [43]. The majority of Iranian people are Muslim and follow the principles of Islam. In the Islamic tradition, laws and principles are derived from a divine source and are articulated within the framework of Sharia. Consequently, the foundations of ethics are inherently intertwined with religious precepts and cannot be separated. So, considering the supreme status of humans in Islam, life is sacred, and terminating it under any circumstances is unacceptable [44]. Other studies have also shown that religious people are more committed to moral principles, social, political, and religious laws [45, 46].

Based on our findings, participants first identify ethical issues, then analyze the situation, hesitate to think, and make decisions based on the various aspects of their work when facing ethical dilemmas. This process is known as reflection. In this stage, students utilize various sources, including personal knowledge, knowledge provided by other involved people, academic resources, and previous experiences [47].

Our results revealed that many participants behaved passively when facing ethical issues, were mostly observers or witnesses, and did not actively engage in ethical issues management. Findings from the literature also exhibited that many intern students choose passivity when confronted with ethical dilemmas, opting to remain silent, which aligns with the results of our study [7, 48, 49]. The findings of similar studies support the claim that nursing students often lack both knowledge and skills in moral reasoning. However, some studies present conflicting results [50, 51]. Enhancing skills such as ethical reasoning, sensitivity, and courage in nursing students before their internship course will lead to making the appropriate ethical decisions in facing ethical dilemmas. To achieve this goal, it is necessary to emphasize the critical and outstanding clinical educators’ roles. They can serve as role models for effectively managing ethical dilemmas within the clinical setting [37, 52]. As the future generation of healthcare practitioners, nursing students should possess ethical sensitivity to deliver high-quality, holistic patient care and address ethical challenges. Ethical sensitivity plays a supreme role in identifying and addressing ethical dilemmas. Achieving this also requires empowering professional ethics educators to sensitize students to moral dilemmas.

### An ineffective ethical atmosphere in the clinical environment

Data analysis in this study revealed another main category entitled “An Ineffective Ethical Atmosphere in the Clinical Environment,” characterized by two sub-categories: “Inefficient professional communication of nurses with other people” and “non-educational environment in terms of professional ethics.”

Participants reported experiencing ineffective professional communication by nurses with various individuals, including patients, caregivers, and nursing students. The nurse’s capacity to establish effective therapeutic communication with patients is crucial for enhancing patient satisfaction and the overall quality of nursing care. The findings of some studies have shown that nurses lack appropriate communication skills. This deficiency can be attributed to insufficient training or their underappreciation of the significance of patient-centered therapeutic communication [53, 54]. Internship nursing students also complained about the nurses’ unprofessional communication with them, which is in line with the findings of previous studies [17, 42]. In the clinical setting, the nurses serve as a mirror of the profession’s reality for students and play a significant role in motivating or demotivating them [48]. So, nursing staff can create a positive psycho-social environment for internship students by fostering interactions based on respect and honesty, given their crucial role in shaping the professional identity of nursing students.

The clinical and educational environment should be in harmony with each other to provide a suitable platform for strengthening the standard clinical skills of nursing students. However, the experiences of the internship students in the present study indicate shortcomings, including the gap between academia and the clinical environment, nurses’ poor attention to compliance with care standards, insufficient focus on student education, and an unsafe discharge process. These findings are consistent with prior studies on the experiences of nursing students [17, 34, 55–57]. The gap between education and the clinical setting can lead to confusion and bewilderment among nursing students during their internships. As a result, they may struggle to differentiate between correct and incorrect practices [57, 58]. Internship students may be learning non-standard procedures by observing nursing staff in the clinical environment, which could be considered a part of the hidden curriculum that is often overlooked.

### Limitations

In the present study, an attempt was made to translate and label the findings in a manner that would be acceptable across different cultures. However, we acknowledge the limitations of language, especially in qualitative studies. The nursing culture and ethical atmosphere governing the healthcare system in Iran may differ from other countries. With this in mind, we aimed to describe our methods and findings in a way that would enable other researchers to apply this study to their context. This study specifically focused on exploring the experiences of Iranian nursing internship students (in semesters 7 and 8). It is suggested that a similar study be conducted with students in lower semesters who are engaged in a clinical environment under the guidance of clinical professors. This would provide valuable insights into how these students handle ethical issues in their training.

## Conclusion

Iranian nursing students who participate in internships lack sufficient preparation and ethical maturity, which hinders their ability to address ethical principles in clinical settings. This often results in confusion and passive behavior when they face ethical issues. Considering the non-educational atmosphere of the clinical environment and deficiencies in the educational structure, it seems imperative to review and reassess the curriculum of professional ethics in nursing. Additionally, conducting surveys to assess the current state of nursing professional ethics training and the educational environment can provide valuable insights into the challenges and gaps that exist. This information can then be used to develop appropriate strategies to bridge the gap between theory and practice in nursing education.

## Electronic supplementary material

Below is the link to the electronic supplementary material.

Supplementary Material 1

## Data Availability

The data supporting the findings of this study are available from the authors, but restrictions apply to their availability. The data were used under the supervision of Golestan University of Medical Sciences for the current study and are not publicly available. However, the data can be obtained from the authors upon reasonable request and with permission from Golestan University of Medical Sciences.
