# Supplementary material for: Experiences of internship nursing students in confronting ethical issues: a qualitative study
Source: BMC Med Ethics. 2025 Jul 11;26:97. doi: 10.1186/s12910-025-01254-w (PMC12247464; doi:10.1186/s12910-025-01254-w)
Supplement: Supplementary file 1 — Supplementary Material 1 [file 12910_2025_1254_MOESM1_ESM.docx]

**interview Guide**

**1. Primary Questions**

- Can you describe one of your work shifts in the clinical environment (internship) for me?
- what do you do to take care of the patient from the moment you enter the ward?

**2. Main Questions**

- During your internship in various hospital wards, have you encountered any issues that you found to be of ethical significance?
- Have you encountered situations where you had to choose between several options? such as whether to be honest with a patient or not. What would you do if you encountered it?

**3. Probing Questions**

Based on the experiences of the participants, exploratory questions were asked. including:

- Please explain more.
- Please clarify the issue.
- Can you give an example**?**

**4. Terminal Phase**

At the end of the interview, to make sure that the participants have expressed all their experiences, the question was asked:

- "Is there anything else you want to say?" I am eager to hear your experiences.
